# Wear and Tear; or, Hints for the Overworked

**Published:** 1887-06

**Authors:** 


**Wear and Tear; or, Hints for the Overworked.** By S. WEIR MITCHELL, M. D., LL.D., Harv., Member of the National Academy of Sciences, President of the College of Physicians of Philadelphia, etc. Fifth edition, thoroughly revised. Philadelphia. 1887.

This is an admirable work to put in the hands of the mentally overworked—and who of the many thousand of brain-workers is not? This little volume, now in its fifth edition, has done much to correct the evils of our American system of constant overwork. It aims to correct the abuses in methods of education; the errors in our dress and habits of living. So many hints are given in this little work that are very valuable, it is the duty of every physician to read it and place it in the hands of his patients. The present edition is superior in many respects to the former numbers.

